# Relationship between surgical rhinoplasty and Demodex infestation: a case-control study

**DOI:** 10.55730/1300-0144.5743

**Published:** 2023-11-11

**Authors:** Abdullah DEMİRBAŞ, Dilek BAYRAMGÜRLER, Emrah Kağan YAŞAR, Muhammed ADAK, Can İlker DEMİR

**Affiliations:** 1 Department of Dermatology, Faculty of Medicine, Kocaeli University, Kocaeli, Turkiye; 2 Department of Plastic, Reconstructive and Aesthetic Surgery, Faculty of Medicine, Kocaeli University, Kocaeli, Turkiye; 3 Department of Dermatology, Midyat State Hospital, Mardin, Turkiye

**Keywords:** Demodicosis, rhinoplasty, surgery

## Abstract

**Background/aim:**

Human Demodex mites are parasites that live in the pilosebaceous unit and can cause demodicosis. While demodicosis may occur as a primary skin disease, it may also result from immunosuppression and topical or systemic immunosuppressive therapies. Surgical rhinoplasty is one of the most commonly performed cosmetic procedures, and it is the cause of a variety of cutaneous complications, particularly acne, as it affects the skin’s adnexal structures. Thus, this study aimed to investigate whether the cutaneous changes in surgical rhinoplasty patients render them vulnerable to Demodex infestation.

**Materials and methods:**

Individuals who had undergone rhinoplasty (patients) and age- and sex-matched healthy volunteers (controls) were included in this prospective case-control study. To determine the Demodex density, samples were collected from the malar and nasal regions of both the patients and controls using the standard superficial skin biopsy method.

**Results:**

A total of 50 rhinoplasty patients and 50 healthy controls were enrolled in the study. The Demodex density on the nose was significantly higher in the rhinoplasty patients (p = 0.0001). Furthermore, the frequency of xerosis and pustules was significantly higher in the rhinoplasty patients compared to the control group (p = 0.046 and p = 0.001, respectively).

**Conclusion:**

Surgical rhinoplasty may be a risk factor for demodicosis, and patients will recover faster after surgery with proper diagnosis and treatment.

## 1. Introduction

Demodicosis may occur as a primary cutaneous condition caused by Demodex mites (Demodex folliculorum and Demodex brevis), as well as secondary to local or systemic immunosuppression. D. folliculorum and D. brevis are common commensal organisms of the human pilosebaceous unit. These mites are most commonly found on the face, scalp, and upper chest [1]. D. folliculorum typically inhabits the follicular infundibulum, whereas D. brevis inhabits the sebaceous ducts and meibomian glands [2,3]. When the number of Demodex mites increases or infests the dermis, several diseases may emerge, such as perioral dermatitis, papulopustular rosacea, pityriasis folliculorum, scabies-like eruptions, demodicosis gravis, and blepharitis [1–3].

Surgical rhinoplasty is one of the most common cosmetic aesthetic surgeries to improve facial appearance by altering the nasal skeletal contour and the skin-soft tissue covering its structural framework [4]. As with all surgical procedures, major and minor complications may develop after rhinoplasty. The major complication rate is reported to be approximately between 1.7% and 18% and can be classified as hemorrhagic, infectious, traumatic, functional, and aesthetic. Minor cutaneous and soft tissue complications are more common due to factors such as inflammation in adnexal skin structures caused by rhinoplasty-related damage, stress, an increase in the corticotropin-releasing hormone, and the application of adhesive tape. These complications include allergic and irritant contact dermatitis, seborrhea, acne exacerbation, rosacea, periorbital hyperpigmentation, persistent nasal cutaneous erythema, and telangiectasias [5–12].

Demodex colonization, which can cause acne-like lesions, has not been investigated in previous studies. Thus, this study aimed to determine whether cutaneous changes in rhinoplasty patients make them susceptible to Demodex infestation.

## 2. Materials and methods

### 2.1. Subjects

This single-center prospective case-control study included patients who had undergone surgical rhinoplasty and age- and sex-matched healthy volunteers who applied to our outpatient clinic between January 1st and June 1st, 2022. The procedure was conducted by 2 distinct surgeons within our department specializing in Plastic Reconstructive and Aesthetic Surgery. For each patient, 1 g of cefazolin administered intravenously as an antibiotic prophylaxis and 8 mg of dexamethasone administered intravenously for its antiedema effect were routinely employed immediately prior to the surgical procedures.

Demographic parameters of the patients such as age, sex, and Fitzpatrick skin phototype (I–VI) [13] were recorded. Furthermore, dermatological symptoms and findings, including the feeling of dryness, burning-stinging sensation, itching, roughness-redness, acne-like rash, erythema, xerosis, follicular spinous protrusion, papule, pustule, and nodule were evaluated [1].

### 2.2. Inclusion and exclusion criteria

Patients between the ages of 18 and 65 who had undergone rhinoplasty at least 6 months prior (as the rhinoplasty group), as well as healthy volunteers between the ages of 18 and 65 without a history of disease or rhinoplasty surgery (as the control group), were included. Prior to the surgical procedure, the individuals who underwent rhinoplasty exhibited no presence of Demodex. The exclusion criteria were as follows: pregnancy or breastfeeding, use of any topical treatment on the face during the previous month, congenital or acquired immune deficiency, and/or use of antiparasitic medicine within the last month.

### 2.3. Standard superficial skin biopsy (SSSB)

To determine the Demodex density, samples were collected from the malar and nasal regions of each participant using the SSSB method [14]. Before the sampling process, the skin of each participant was cleaned with ethanol to improve the ability of the glass slide to adhere to the skin. A drop of cyanoacrylate was applied in the center of the marked area on the opposite surface of the slide. Slides containing cyanoacrylate drops were gently put on the right and left cheeks and nose for 1 min, ensuring contact between the slide and the skin, and then slowly lifted. Biopsy samples were examined by the principal investigator at 10X and 40X magnification under a light microscope (Leica DM500 binocular microscope Danaher Inc. Washington, DC, USA) after dripping oil immersion. If the number of Demodex mites in the sample material was more than 5/cm^2^, it was found to be significant for demodicosis (Figure) [14]. Determination of the participants’ final Demodex density in 1 cm^2^ was calculated using the sample with the highest number of Demodex among the 2 samples collected from them.

### 2.4. Statistical analysis

IBM SPSS Statistics for Windows 23.0 (IBM Corp., Armonk, NY, USA) was used for the statistical analysis. The descriptive statistics were expressed as the mean and median, and number and percentage. The Kolmogorov–Smirnov test was used to evaluate the normality of the data distribution. Pearson’s chi-squared analysis was used to evaluate the relationships between the categorical variables. p < 0.05 was accepted as statistically significant.

### 2.5. Ethics approval

All of the procedures were carried out following the ethical principles set forth in the Helsinki Declaration, and the study was approved by an Institutional Review Board (Decision date and number: 2021/363). All of the participants provided written informed consent.

## 3. Results

The study included 50 rhinoplasty patients (37 females, 13 males) and 50 age- and sex-matched healthy controls (32 females, 18 males) (Table 1).

The mean ages of the patients and controls were 25.5 ± 4.8 and 24.8 ± 5.2 years, respectively (p = 0.425). In regard to the Fitzpatrick skin classification, 5 (10%) patients were type 1, 8 (16%) were type 2, 24 (48%) were type 3, and 13 (26%) were type 4 (Table 1).

The most common preoperative (preop) symptom reported by the patients was the feeling of dryness (n = 10, 20%), followed by acne-like rashes (n = 8, 16%), roughness-grating sensation (n = 7, 14%), burning-stinging (n = 5, 10%), and pruritus (n = 1, 2%) (Table 2). However, the most common postoperative (postop) symptom reported by the patients was acne-like rashes (n = 22, 44%), followed by itching (n =18, 36%), the feeling of dryness (n = 14, 28%), burning-stinging (n = 5, %10), and roughness-grating sensation (n = 4, 8%) (Table 2). The frequency of xerosis and pustules in the dermatological examination findings was statistically significantly higher in the rhinoplasty patients compared to the control group (p = 0.046 and p = 0.001, respectively) (Table 2). The occurrence of erythema, follicular spinous protrusions, papules, and nodules did not show any statistical significance between the groups (p > 0.05) (Table 2).

The nasal SSSB was positive in 19 (38%) of the rhinoplasty patients and 4 (8%) of the healthy controls. The malar SSSB was positive in 8 (16%) of the rhinoplasty patients and 7 (14%) of the healthy controls. When the rhinoplasty group was compared to the control group, the difference in the nasal Demodex density was statistically significant, while the malar Demodex density was not (p = 0.0001 and p = 0.779, respectively) (Table 3).

There was no significant relationship between the nasal Demodex density and the preop and postop dermatological symptoms in the rhinoplasty group (p > 0.05) (Table 4).

## 4. Discussion

The Demodex mite is an obligatory human ectoparasite found in seborrheic areas such as the nasolabial folds, periorbital regions, and, less frequently, the upper and medial chest and back [15]. Demodex infestations are typically asymptomatic and may only be pathogenic when high densities are present, or the immune system is compromised. Primary or secondary immunosuppression development may play a role in the transition from clinically nonobvious mite colonization to dermatoses [16,17]. T-cell defects are most likely the underlying cause of primary immunosuppression [1,15–18]. Secondary immunosuppression that predisposes to demodicosis is caused by corticosteroid therapy, chemotherapy, or immunosuppressed diseases such as malignant neoplasms or HIV infection. Several studies have investigated the underlying cause of secondary demodicosis and the association between demodicosis and a variety of systemic diseases [1,15,19–23].

In a case study, the Demodex density was found to be increased in pediatric patients with Langerhans cell histiocytosis, cerebral palsy, and epilepsy. It was also reported that immunosuppression has been associated with the development of demodicosis [24]. In another study, it was reported that secondary demodicosis developed in patients receiving systemic immunosuppressive therapy due to kidney transplantation and that it may be related to the underlying immunosuppressive condition [25]. Previous studies have indicated that the Demodex density increases in patients with AIDS, diabetes, hemodialysis, Hashimoto’s thyroiditis, end-stage renal disease, and after allogeneic stem cell transplantation [26–32].

Due to the impact on adnexal cutaneous structures, blood vessels, and nerves, surgical rhinoplasty operations can result in skin conditions such as acne, ecchymosis, desquamation, hyperpigmentation, loss of brows, scars at the operation site, skin necrosis, telangiectasia, and seborrhea [5–10]. A few studies have been conducted to investigate acne formation following rhinoplasty. Nemati et al. revealed that mild acne developed after rhinoplasty in 42.9% of patients, and acne exacerbation occurred in 27% of patients within 1 month of surgery [6].

Furthermore, Koç et al. found an increase in postop seborrheic dermatitis and acne severity scores in septorhinoplasty patients [7]. Another study found that 60% of 147 patients who had rhinoplasty operations experienced acne recurrence and exacerbation [8]. Damage to the adnexal skin structures during the operation is thought to cause inflammation, an increase in the number of Propionibacterium acnes in the microflora of the sebaceous follicles, and eventually, follicle rupture and acne. Furthermore, stress, an increase in the corticotropin-releasing hormone, the use of adhesive tape, not washing the face frequently in the early period, as well as externally applied corticosteroids that are sometimes administered before surgery, and systemic antibiotics used to reduce the risk of infection, have all been associated with acne formation [6–8].

Various studies have also shown that, unlike seborrhea, surgical rhinoplasty can cause severe dryness and allergic and irritant contact dermatitis on the faces of patients [4,5,11,12].

Turan et al. stated that when the faces of their acne and rosacea patients were acidic, dry, or very dry, this facilitated the development of demodicosis [33]. In the present study, the nasal Demodex density was significantly higher in the rhinoplasty group compared to the control group. Furthermore, the frequency of xerosis and pustules was significantly higher in the rhinoplasty group than in the control group. The topical corticosteroids used to reduce edema, stress, and the fact that the patients’ faces become drier may be factors that increase the Demodex colonization density.

Demodicosis can cause rashes similar to acne lesions. It should be considered alongside acne and rosacea diseases in pustules in patients who have undergone surgical rhinoplasty. In this situation, with proper diagnosis and treatment, postop patients may experience less anxiety and stress, and recover faster.

To the best of our knowledge, this is the first study to examine the relationship between surgical rhinoplasty and D. folliculorum colonization density. The relatively small sample size was the most significant limitation of the study. Therefore, further research with larger sample sizes is required to validate the findings of the study.

## Figures and Tables

**Figure:**
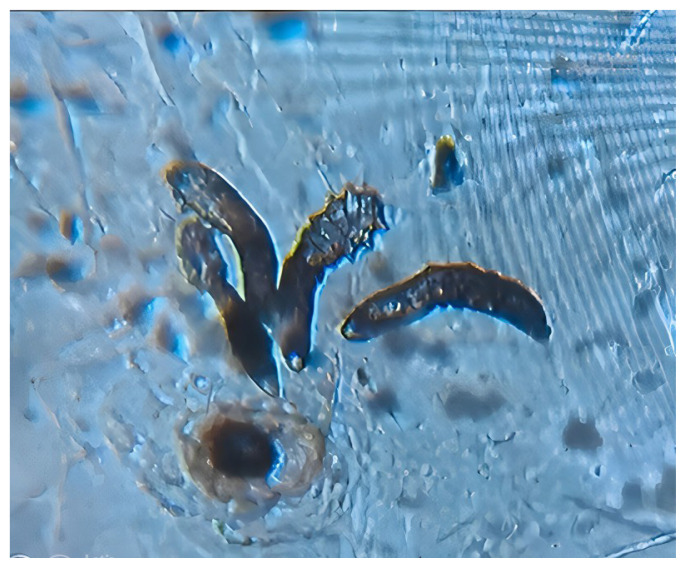
Observation of the Demodex mites using a light microscope.

**Table 1:** Demographics and general features of the rhinoplasty and the control groups.

	Rhinoplasty group (n = 50)	Control group (n = 50)	p-value
Age (years), mean ± SD	25.5 ± 4.80	24.8 ± 5.20	0.425
Male/female, n (%)	13 (26%)/37 (74%)	18 (36%)/32 (64%)	0.280
Fitzpatrick skin type, n (%) Type 1	5 (10%)	1 (2%)	0.365
Type 2	8 (16%)	8 (16%)	
Type 3	24 (48%)	29 (58%)	
Type 4	13 (26%)	12 (24%)	

**Table 2:** Preop and postop dermatological symptoms in the rhinoplasty group.

	Rhinoplasty group (n = 50)	
	**+**	−
Preop symptoms on/around the face, n (%)		
Itching	1 (2%)	49 (98%)
Burning-stinging	5 (10%)	45 (90%)
Roughness-grating sensation	7 (14%)	43 (86%)
Acne-like rash	8 (16%)	42 (84%)
Feeling of dryness	10 (20%)	40 (80%)
Postop symptoms on/around the face, n (%)		
Itching	18 (36%)	32 (64%)
Burning-stinging	5 (10%)	45 (90%)
Roughness-grating sensation	4 (8%)	46 (92%)
Acne-like rash	22 (44%)	38 (76%)
Feeling of dryness	14 (28%)	36 (72%)

+ Present, − absent.

**Table 3:** Evaluation of the dermatological examination findings and nasal and malar Demodex densities in the rhinoplasty group after surgery and the control group.

	Rhinoplasty group (n = 50)		Control group (n = 50)		p-value
	+	−	+	−	
Dermatological examination findings, n (%)	11 (22%)	39 (78%)	5 (10%)	45 (90%)	0.102
Erythema					
Xerosis	14 (28%)	36 (72%)	6 (12%)	44 (88%)	**0.046**
Follicular spinous protrusion	6 (12%)	44 (88%)	2 (4%)	48 (96%)	0.14
Papule	16 (32%)	34 (74%)	9 (18%)	41 (82%)	0.106
Pustule	20 (40%)	30 (60%)	6 (12%)	44 (88%)	**0.001**
Nodule	0 (0%)	50 (100%)	0 (0%)	50 (100%)	**-**
Nasal Demodex density, n (%)	19 (38%)	31 (62%)	4 (8%)	46 (92%)	**0.0001**
Malar Demodex density, n (%)	8 (16%)	42 (84%)	7 (14%)	43 (86%)	0.779

**Table 4:** Relationship between the nasal Demodex density in the rhinoplasty group and the dermatological symptoms before and after surgery.

		Nasal Demodex density in the rhinoplasty group after surgery		p-value

		+ (n = 19)	− (n = 31)	

Preop itching	+	1	0	0.197
	
	−	18	31	

Preop burning-stinging	+	17	28	0.923
	
	−	2	3	

Preop roughness-grating sensation	+	3	4	0.775
	
	−	16	27	

Preop acne-like rash	+	3	5	0.974
	
	−	16	26	

Preop feeling of dryness	+	3	7	0.56
	
	−	16	24	

Postop itching	+	9	9	0.19
	
	−	10	22	

Postop burning-stinging	+	2	3	0.923
	
	−	17	28	

Postop roughness-grating sensation	+	2	2	0.606
	
	−	17	29	

Postop acne-like rash	+	11	11	0.121
	
	−	8	20	

Postop feeling of dryness	+	8	6	0.082
	
	−	11	25	
